# *De novo* transcriptome profiling uncovers a drastic downregulation of photosynthesis upon nitrogen deprivation in the nonmodel green alga *Botryosphaerella sudeticus*

**DOI:** 10.1186/1471-2164-14-715

**Published:** 2013-10-19

**Authors:** Deying Sun, Jiaqi Zhu, Lei Fang, Xin Zhang, Yvonne Chow, Jianhua Liu

**Affiliations:** Systems Biology, Genome Institute of Singapore, 60 Biopolis Street, #02-01, Singapore, 138672 Singapore; Industrial Biotechnology, Institute of Chemical and Engineering Sciences, Singapore, 627833 Singapore

**Keywords:** Next-generation sequencing, De novo assembly, Transcriptome profiling, Metabolic pathways, *B. sudeticus* UTEX2629

## Abstract

**Background:**

Neutral lipid storage is enhanced by nitrogen deprivation (ND) in numbers of green microalgal species. However, little is known about the metabolic pathways whose transcription levels are most significantly altered following ND in green microalgae, especially the nonmodel species.

**Results:**

To start gaining knowledge on this, we performed transcriptome profiling of the nonmodel green microalga *Botryosphaerella sudeticus* cells in response to ND. Transcriptome of *B. sudeticus* is *de novo* assembled based on millions of HiSEQ short sequence reads using CLC Genomics Workbench software. The resulting non-redundant ESTs are annotated based on the best hits generated from the BLASTX homology comparison against the “best” proteins in the model microalgae *Chlamydomonas reinhardtii* and *Chlorella variabilis.* By using a pathway-based approach according to KEGG databases, we show that ESTs encoding ribosomal proteins and photosynthetic functions are the most abundantly expressed ESTs in the rapidly growing *B. sudeticus* cells. We find that ESTs encoding photosynthetic function but not the ribosomal proteins are most drastically downregulated upon ND. Notably, ESTs encoding lipid metabolic pathways are not significantly upregulated. Further analyses indicate that chlorophyll content is markedly decreased by 3-fold and total lipid content is only slightly increased by 50%, consistent with the transcriptional profiling. On the other hand, carbon content and photosynthesis efficiency are only marginally decreased by 7% and 20%, respectively, indicating that photosynthesis is only slightly reduced upon drastic downregulation of photosynthetic ESTs and chlorophyll content upon ND. In addition, TAG content is found to be greatly increased by 50-fold, though total lipid content is only slightly increased by 1.5-fold.

**Conclusions:**

Taken together, our results suggest that light-harvesting proteins and chlorophylls are in excess in *B. sudeticus*. Degradation of excess photosynthesis proteins is most likely a mechanism for recycling of nitrogen-rich molecules to synthesize new proteins for preparation of gametogenesis and zygospore formation in adaptation and survival upon ND. Furthermore, our analyses indicate that TAG accumulation is largely attributed to the modification of other pre-existing lipid molecules, rather than de novo synthesis. We propose that this is likely an evolutionarily conserved mechanism in many green microalgae species.

**Electronic supplementary material:**

The online version of this article (doi:10.1186/1471-2164-14-715) contains supplementary material, which is available to authorized users.

## Background

Economic growth is often accompanied by the increase in energy consumption. Depletion of fossil fuel reserves and rising crude oil prices prompt renewed interest in algae biofuel research. Green microalgae are unicellular photosynthetic organisms capable of converting photon energy into chemical energy and assimilating carbon dioxide to form glucose. It has been proposed that microalgae farming could avoid competing with food crops for arable land and fresh water and the energy yield from algae per acre per year would be much higher than other crops [1–3]. In addition, it mitigates greenhouse gas emission. However, current microalgal species and cultivation methodologies are thought to hamper production of algae-based energy in an economically viable manner [1, 4].

Many studies have focused on the enhancement of lipid contents in microalgae. Nitrogen deprivation (ND) is one of the most widely used methods to increase lipid storage in algae [4–10]. It is known that ND triggers gametogenesis and zygospore formation in the model green microalga *C. reinhardtii* [11]. Zygospores can survive under harsh conditions without nutrients for long period of time and ready to revive through germination when external nutrients become available. Storage molecules such as starch and fats (e.g., triacylglycerol or TAG) are the major source of energy essential for spore germination before they are capable of assimilating external nutrients. Therefore, enhancement of lipid storage following ND is at the cost of cell growth arrest and differentiation.

Global transcriptional profiling of microalgal cells in response to ND using next-generation sequencing (or NGS) technologies allows identification of gene regulatory networks involved in adaptation and survival [7, 12]. Global transcriptional profiling indicates that alteration of lipid metabolic pathways is complex: enhancement of TAG accumulation could be a result of recycling of membrane lipids and *de novo* glycerolipid biosynthesis [7]. Comparative transcriptome analysis with other algal species may allow gaining insight into molecular mechanisms underlying the metabolic pathways involved in growth arrest and biosynthesis of storage molecules.

Currently, a number of microalgal genome such as *Chlamydomonas reinhardtii* and *Chlorella variabilis* are fully sequenced and comprehensively annotated [13, 14]. Additionally, assembly of genomes and transcriptomes from the millions of short sequence reads generated by NGS technologies has been greatly aided by the use of de Bruijn graph-based sequence-alignment algorithms [15–18]. Several green microalgal transcriptomes have been assembled without reference genomes by using these methodologies [19–21]. However, most of these analyses are primarily focused on gene discovery and metabolic pathway reconstitution.

The green microalga *Botryosphaerella sudeticus* (UTAX2629) has been previously isolated from a culture of the hydrocarbon-rich slow-growing microalga *Botryococcus braunii* as a contaminant [22]. Phylogenetic analysis based on rDNA sequence indicates that *B. sudeticus* is more related to *Characiopodium hindakii* rather than *B. braunii* [23]. Observation of gametes leads to the suggestion that *B. sudeticus* may have sexual life cycle [24]. We showed that the maximal cell density of *B. sudeticus* cultures was 2-fold of that of *C. reinhardtii* under conditions of phototrophic growth, suggesting a useful candidate for algal biomass production. Gametogenesis and lipid accumulation were induced by ND. To investigate transcriptional alteration of metabolic pathways following ND, we performed global transcriptional profiling of *B. sudeticus* cells in response to ND based on the transcriptome assembled without reference genome. By using a pathway-based approach, we show that ESTs encoding photosynthetic function and ribosomal proteins are the most abundant ESTs in rapidly growing *B. sudeticus* cells. Upon ND, photosynthetic function-encoding but not ribosomal protein-encoding ESTs are drastically downregulated, suggesting a programmed cellular response for differentiation. This transcriptional response to ND is found to be conserved in *C. reinhardtii*, possibly in most green microalgae.

## Results

### Gametogenesis and lipid accumulation are enhanced by nitrogen deprivation (ND) in B. sudeticus

**Figure 1:**
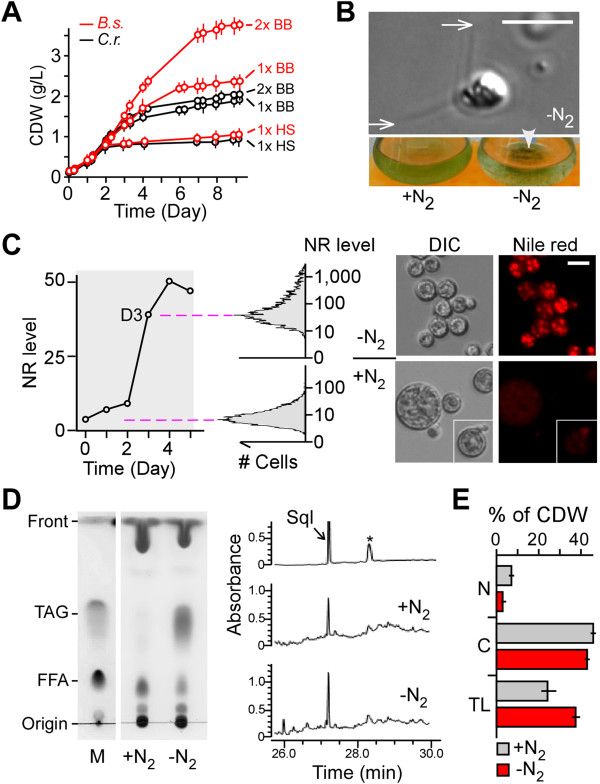
**Characterization of growth, gametogenesis, and lipid content in** ***B. sudeticus*** **. (A)** *B. sudeticus* culture reaches relatively high maximal cell density. Growth curves of *B. sudeticus* (in red) and *C. reinhardtii* (in black) in various media indicated are shown. X- and Y-axis indicate time (in day) and cell dry weight (CDW; in g/L), respectively. HS and BB stand for High Salt and Bold-modified Bristol media, respectively. Error bars represent the standard deviation of triplicate samples. **(B)** Gametogenesis is induced by ND in *B. sudeticus*. Upper panel shows a gamete whose tip of flagella is denoted by an arrow. Bottom panel shows part of conical flasks in which sedimented cell aggregates are denoted by an arrow-head. Medium with nitrogen (+N_2_) or without nitrogen (-N_2_) is indicated. **(C)** Lipid accumulation is enhanced by ND in *B. sudeticus*. Left panel shows the increased intensity of Nile red signal in cells after ND. X- and Y-axis indicate the time (in day) and Nile red signal level (in arbitrary units), respectively. Nile red signal level of cell samples are based on the median level derived from FACS analysis as shown in the mid panel. Right panel shows the Nile red stained cells prior to (+N_2_) and after (-N_2_) ND. **(D)** Level of triacylglycerol (TAG) and squalene is increased in *B. sudeticus* following ND. Left panel shows the TLC analysis and right panel shows the GC-MS analysis (MS result is not shown) of total lipid in *B. sudeticus* prior to (+N_2_) and after (-N_2_) ND. **(E)** Contents of nitrogen (N), carbon (C), and total lipid (TL). Bar-plots show the level of N, C, and TL against CDW in nitrogen-replete (+N_2_) and nitrogen-starved (-N_2_) cells.

*B. sudeticus* (UTEX2629) was often found to overgrown in cultures of *B. braunii* (UTEX572) (Sun and Liu, unpublished data). It was partly because of the slow growth rate of *B. braunii*. To investigate how well *B. sudeticus* grew, we tested its phototrophic growth in media of HS [25], BB [26], and 2x BB in shaking flask with bubbling of CO_2_ under constant light (i.e., 250 µmol photon m^-2^ sec^-1^). We compared the growth curve of *B. sudeticus* with that of *C. reinhardtii*, a best-studied green microalga. The growth rate and the maximal cell density of *B. sudeticus* and *C. reinhardtii* were very similar in HS medium (Figure 1A). On the other hand, though the growth rate of *B. sudeticus* remained to be similar as that of *C. reinhardtii*, the maximal cell density of *B. sudeticus* was increased by ~25% in 1xBB medium and nearly by 100% (or 2-fold) in 2x BB medium compared to that of *C. reinhardtii*. This result suggests that *B. sudeticus* is a useful strain for potential microalgal biomass production.

We subsequently investigate if gametogenesis would be induced by ND. We noted that there were ~1% of gametes in log-phase growth culture (Figure 1B; upper panel). On the other hand, ~10% of gametes were found three days after ND. Though we did not obtain the cell-cell fusion event in EM analysis (Data not shown), aggregation and precipitation of cells upon ND was apparent (Figure 1B; bottom panel). Hence, we concluded that ND induced gametogenesis in *B. sudeticus*.

To examine whether ND would enhance lipid storage, nitrogen-deprived (ND) cells were stained with a lipophilic dye Nile red and subjected to FACS analysis (Figure 1C). Average level of Nile red signals in cells 3 days after ND was increased by 40-fold compared to that prior to ND. TLC and GC-MS analyses indicated that TAG and squalene (SQL) contents (i.e., % of CDW) were increased by ~50-fold and 2.5-fold compared to that of nitrogen-replete cells (i.e., TAG, 3.74±0.14% versus 0.08±0.01% of CDW, p-value = 1.5e-06; SQL, 0.15±0.01 versus 0.06±0.01% of CDW, p-value = 6.4e-05), respectively (Figure 1D; Additional file 1: Table S8). Notably, total lipid of the early log-phase cells exhibited the relatively low level of TAG content.

Actively growing *B. sudeticus* cultures were maintained by subculturing or 8-fold dilution in fresh BB+N medium prior to exhaustion of nitrate in the medium (i.e., 3.8±0.3 mg/L nitrate at day 3 prior to dilution/ subculturing). We noted that the cell mass continued to increase under ND conditions (i.e., CDW from 0.14±0.03 g/L at day 0 to 0.94±0.06 g/L at day 3 under ND conditions). To examine the alteration of nitrogen (N) and carbon (C) contents in cells after ND, we performed elemental analysis of N and C in cells. It was clear that N contents in the ND cells was greatly dropped by 2.5-fold compared to that of nitrogen-replete cells (i.e., 2.8±0.09% versus 7.14±0.16% of CDW, p-value = 2.16E-06) (Figure 1E). On the other hand, the C content in the ND cells was only slightly reduced compared to that of nitrogen-replete cells (i.e., 42.7±0.31% versus 45.9±0.46% of CDW). This result suggests that carbon assimilation is not strongly affected upon ND for 3 days. While TAG content were drastically increased by 50-fold upon ND stress (i.e., 3.47±0.14% versus 0.08±0.01% of CDW; p-value = 1.5e-06), total lipid (TL) content in ND cells was only slightly increased by 1.54-fold compared to that of nitrogen-replete cells (i.e., 37.37±1.42% versus 24.21±2.71% of CDW). This result indicates that majority of the storage TAG molecules in ND cells are accumulated through the modification of pre-existing lipids, rather than de novo synthesis.

### *De novo* assembly and annotation of the *B. sudeticus* transcriptome

**Figure 2:**
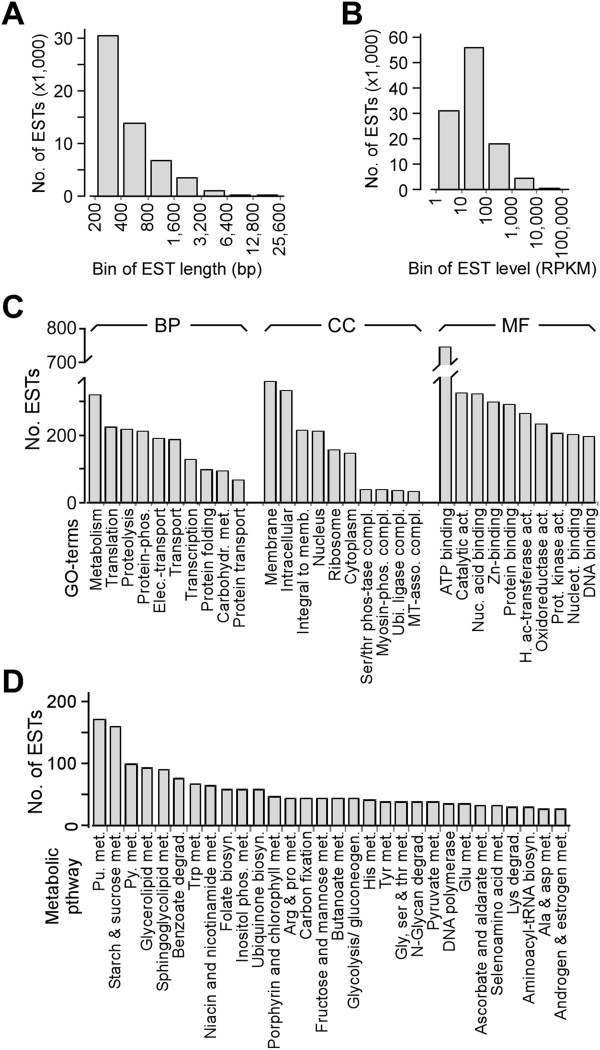
**Characteristics of the** ***B. sudeticus*** **transcriptome.** A total of 56 thousand ESTs is assembled based on 53 million paired-end reads (90 bp in length), of which, ~7,600 ESTs were annotated. **(A)** Length distribution of all ESTs. X- and Y-axis indicate the binned EST length and the number of ESTs in each bin, respectively. **(B)** Level distribution of all ESTs. Levels are normalized to RPKM. X- and Y-axis indicate the binned EST level (in RPKM) and the number of ESTs in each bin, respectively. **(C)** List of GO-terms that are associated with ESTs annotations. Top 10 GO-terms in each category are shown. X-axis indicates the GO-terms and Y-axis indicates the number of ESTs associated with the GO-terms. BP, CC, and MF stand for biological process, cellular component, and molecular function, respectively. **(D)** List of KEGG metabolic pathways that are associated with ESTs annotations. Top 30 metabolic pathways are listed. The display is identical to **(C)**.

For analysis of transcriptional profiling of *B. sudeticus* cells in response to ND, we first wanted to assemble the transcriptome without reference genome. For this reason, cDNA libraries were constructed from the ND and nitrogen-replete cells. A total of 52,000,000 paired-reads (90-bp in length) pooled from both growth conditions were subjected to *de novo* transcriptome assembly using the CLC Genomics Workbench software (see Methods). To this end, 56,000 non-redundant contigs/ scaffolds/ ESTs (ESTs were used hereafter) were generated. Length of ESTs ranged from 200 to 15,487 bps with a median of 369 bps or average of 660 bps (Figure 2A). Approx. 90% of all reads in either growth condition was mapped to the ESTs. Absolute level of ESTs was normalized to RPKM (*R*eads *P*er *K*ilobase per *M*illion mapped reads) ranged from 0.09 to 17,293 with a median of 2.21 (Figure 2B).

*B. sudeticus* ESTs were annotated based on the best-hit proteins generated by sequence homology comparison using the Basic Local Alignment Search Tool BLASTX suite (http://blast.ncbi.nlm.nih.gov) against the “best” proteins in the model green microalgae *C. reinhardtii* and *C. variabilis* [13, 14] with a cutoff expectation-value of 1E-06. As a result, a total of 7,625 (13.6%) non-redundant ESTs in *B. sudeticus* were annotated, of which, 6,559 ESTs had best-hits in *C. reinhardtii* and 1,066 in *C. variabilis* [13, 14] (Additional file 2: Table S1).

Gene Ontology (GO) [27] and KEGG Ortholog (KO) [28] annotations that associated with the best-hit proteins in *C. reinhardtii* and *C. variabilis* were extracted for transcriptome analysis in *B. sudeticus*. Of a total of 7,625 annotated ESTs, 4,860 were found to be associated with at least one of the 2,122 GO-terms (i.e., 433 terms in Biological Processes, 152 terms in Cellular Components, and 1,537 terms in Molecular Functions). GO terms associated with the highest number of ESTs in three categories were metabolism (322 ESTs; biological process), membrane (361 ESTs; cellular component), and ATP-binding (744 ESTs; molecular function). The top 10 GO-terms in three categories were shown in Figure 2C (Additional file 3: Table S2).

Out of 7,625 ESTs, 1,129 were found to be associated with at least one of the 114 KEGG metabolic pathways [28]. Top 30 KEGG metabolic pathways were shown in Figure 2D (Additional file 4: Table S3). We took a pathway-based approach to study the coherence of ESTs absolute abundances in the rapidly growing cells and the coherence of ESTs abundance alterations (or ratios) upon ND in *B. sudeticus* (see below).

### Overview of the pathway-based transcriptome analysis

Based on KEGG databases, we focused on the coherence analysis of the absolute abundance (i.e., steady-state level in nitrogen-replete cells) and relative abundance (i.e., ratio between levels in ND and nitrogen-replete cells) of a subset of the metabolic pathway-associated ESTs. We assumed that co-regulated basal or constitutive transcription of the pathway-associated ESTs would display a high consistency in EST levels in rapidly growing cells. Likewise, co-regulated transcriptional alteration in response to ND would exhibit a high coherence of the pathway-associated EST ratios between levels in ND and nitrogen-replete cells.

We used the occurrence density of the pathway-associated ESTs in a range that contained the first 50%, middle 50%, or second 50% of the ESTs based on ranks to approximate the transcriptional coherence of a given metabolic pathways (see Methods). Coherence of the constitutive transcription and ND-induced transcription of the pathway-associated ESTs was determined based on ranks by the absolute abundance and relative abundance or ratio, respectively. A metabolic pathway was designated as a co-transcriptionally regulated (or coherent) pathway if the occurrence density of the pathway-associated ESTs in one of the three half ranges was 2-fold above background and the p-value was less than 0.05 based on ranks by level (i.e., for basal transcription) or ratio (i.e., for transcriptional response). We also tested the occurrence density in the top 10% of the most abundant ESTs based on ranks by level or in the top (or bottom) 10% of the most upregulated (or downregulated) ESTs based on ranks by ratio (e.g., relative occurrence density >2-fold above background and p-value < 0.05) for coherently regulated metabolic pathways (see Methods).

We proposed that coherent pathways were potentially co-transcriptionally regulated. Given that the magnitude of alteration in transcription levels following ND was a function of time, magnitude of change in transcription levels would vary in cells sampled at different time point after stress. On the other hand, coherence of transcriptional response for metabolic pathways would be remained throughout the course of response.

### Coherent transcription of the ribosomal protein-encoding ESTs in B. sudeticus

Absolute level of the 7,625 annotated ESTs in *B. sudeticus* was approximated by RPKM (see Methods). We found that 16 out of top 30 most abundant ESTs encoded putative ribosomal proteins, indicating that RP-encoding ESTs are enriched in the topmost expressed ESTs compared to background (i.e., 53.3% versus 1.29%, p-value < 1.33E-07) in actively growing *B. sudeticus* cells (Table 1). This result is consistent with a notion that protein synthesis is one of the most active functions in actively growing cells [29].

**Table 1:** **List of the top 30 most abundant ESTs in** ***B. sudeticus***

Rk^a^	EID^b^	Level	Ratio	Genome	PID^c^	Description^d^
1	3556	4.03	-0.50	Chlre4	376403	
2	318	3.98	-0.24	Chlre4	322197	RP-S29e, RPS29; small subunit ribosomal protein S29e
3	5	3.81	0.20	Chlre4	395228	
4	36952	3.78	0.16	ChlNC64A	28889	
5	106	3.70	-0.04	Chlre4	155068	
6	57	3.69	0.18	Chlre4	407233	Glucan1,4-alpha-glucosidase.
7	8	3.67	-0.21	Chlre4	195592	RP-S20e, RPS20; small subunit ribosomal protein S20e
8	520	3.65	-0.03	Chlre4	195131	RP-L22e, RPL22; large subunit ribosomal protein L22e
9	127	3.62	0.05	Chlre4	418234	
10	142	3.61	-0.06	Chlre4	128309	RP-L44e, RPL44; large subunit ribosomal protein L44e
11	512	3.56	-0.12	Chlre4	194928	RP-L38e, RPL38; large subunit ribosomal protein L38e
12	181	3.50	0.10	Chlre4	127247	RP-L35Ae, RPL35A; large subunit ribosomal protein L35Ae
13	633	3.45	-0.33	Chlre4	206640	rbcS; ribulose-bisphosphate carboxylase small chain[EC:4.1.1.39]
14	316	3.41	0.00	Chlre4	129742	RP-S21e, RPS21; small subunit ribosomal protein S21e
15	31	3.38	0.05	Chlre4	57302	RP-S25e, RPS25; small subunit ribosomal protein S25e
16	9	3.30	-0.26	Chlre4	166012	RP-L30e, RPL30; large subunit ribosomal protein L30e
17	311	3.26	0.09	Chlre4	191758	RP-L32e, RPL32; large subunit ribosomal protein L32e
18	1207	3.26	0.16	ChlNC64A	144034	
19	114	3.23	-0.33	Chlre4	344365	
20	590	3.19	-0.18	Chlre4	195601	RP-S17e, RPS17; small subunit ribosomal protein S17e
21	1921	3.18	0.21	Chlre4	195587	RP-L11e, RPL11; large subunit ribosomal protein L11e
22	287	3.17	-0.06	Chlre4	292191	
23	282	3.16	-0.41	Chlre4	195585	RP-L10Ae, RPL10A; large subunit ribosomal protein L10Ae
24	50	3.16	-0.84	Chlre4	156131	TC.AMT; ammonium transporter, Amt family
25	20	3.15	0.45	Chlre4	120079	SIRT2, SIR2L2; NAD-dependent deacetylase sirtuin 2 [EC:3.5.1.-]; in linear amides.
26	1000	3.14	-0.26	Chlre4	126853	RP-L37Ae, RPL37A; large subunit ribosomal protein L37Ae
27	480	3.14	-0.16	Chlre4	78109	RP-L31e, RPL31; large subunit ribosomal protein L31e
28	296	3.12	-0.24	Chlre4	36709	
29	278	3.12	0.01	Chlre4	145271	RP-L14e, RPL14; large subunit ribosomal protein L14e
30	545	3.11	-0.07	Chlre4	153674	

Note: a, rank; b, EST ID; c, Protein ID in *C. reinhardtii* or *C. variabilis*; d, EST function based on its best hit protein.

**Figure 3:**
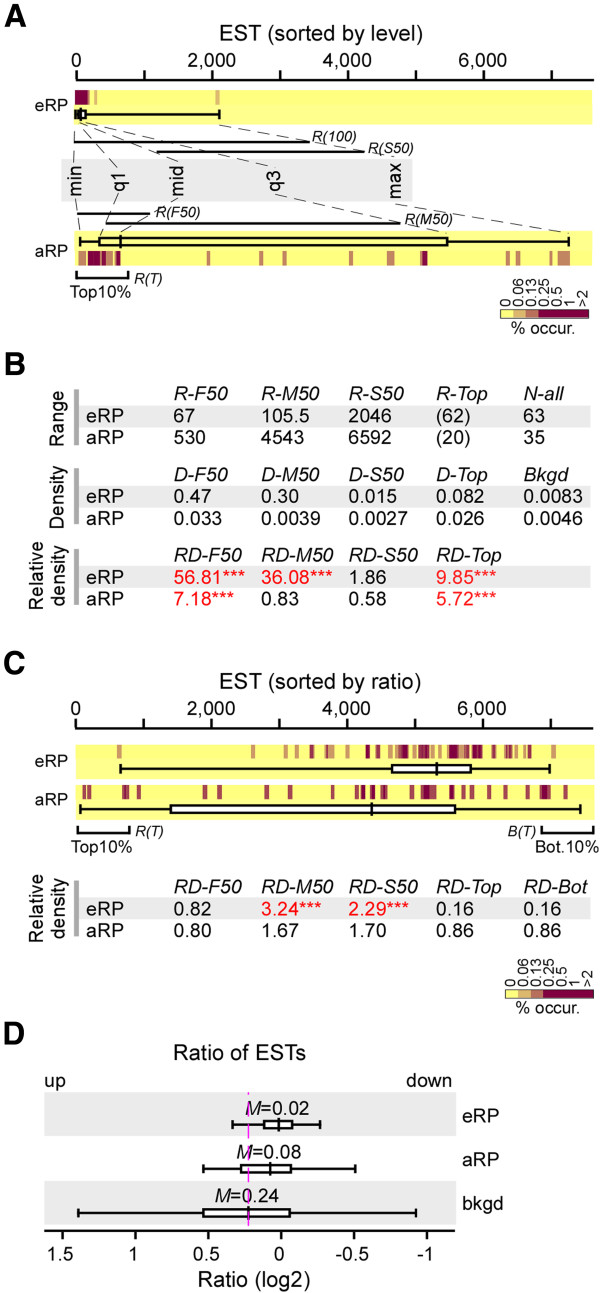
**Coherence of transcription levels in ribosomal protein-encoding ESTs in** ***B. sudeticus*** **. (A)** Distribution of ESTs encoding eRPs and aRPs based on rank by level. The heat-map shows the EST occurrence that is binned by an average sliding window of 11 consecutive ranks and normalized to 1 for each pathway or component. The color key for percent levels is shown at the bottom. The box-plot indicates the minimum (min), first quartile (q1), median (mid), third quartile (q3), and maximum (max) of eRP and aRP EST levels by rank. **(B)** Occurrence densities of eRP and aRP ESTs. Upper table shows the range of ESTs based on rank by level. Four ranges are shown: first 50% (R-F50), middle 50% (R-M50), and second 50% (R-S50) of eRP- or aRP-ESTs and top 10% (R-Top) of most abundant ESTs based on rank by level. Mid table shows the occurrence density of eRP- or aRP-ESTs. Bottom table shows the relative occurrence density in 3 half-ranges containing 50% of the eRP or aRP-ESTs. Coherent transcription or relative density in one of the 3 half-ranges is 2-fold above background and p-value is less than 0.05. Different levels of statistical significance are indicated as triple asterisk (***), double asterisk (**), and single asterisk (*) for p-value < 0.001, < 0.01, and < 0.05, respectively. **(C)** Distribution of eRP- and aRP-ESTs based on rank by ratio. Top panel shows the heat-map and box-plot of eRP- and aRP-EST distribution based on rank by ratio. Bottom table shows the relative occurrence density of eRP- and aRP-ESTs in 3 half-ranges and in top or bottom of the most differentially transcribed ESTs. Levels of statistical significance are indicated as **(B)**. **(D)** Distribution of eRP- and aRP-EST ratios. Change of median ratios (*ΔM*) of eRP- and aRP-ESTs compared to background is indicated.

Cytosolic or eukaryotic ribosomal proteins (i.e., eRPs) are encoded by nuclear genes in green microalgae. On the other hand, chloroplast and mitochondrion or archaea ribosomal proteins (i.e., aRPs) are encoded by both the nuclear and chloroplast or mitochondrion genes, respectively. Transcriptional coordination would be relatively easy to achieve for a group of genes localized in a single genome compared to those in two genomes such as nuclear and chloroplast or mitochondrial genomes. Hence, we hypothesized that the transcription consistency of the eRP-encoding ESTs would be high compared to that of aRP-encoding ESTs. To test this hypothesis, we first determined the relative occurrence density in the 3 half-ranges (i.e., first, middle, and second 50%) of the eRP- and aRP-encoding ESTs (i.e., nuclear encoded aRP genes) based on the rank by abundance (Figures 3A and 3B). It was clear that the relative occurrence densities in the first and middle halves of the eRP-encoding ESTs were much higher than background (i.e., 56.8-fold and 36.1-fold above background; p-value < 0.001), indicating that 75% of the eRP-encoding ESTs displayed a coordinated level of constitutive transcription. On the other hand, the relative occurrence density only in the range of the first 50% of the aRP-encoding ESTs was higher than background (i.e., 7.2-fold above background; p-value < 0.001).

We noted that the occurrence density of both eRP- and aRP-encoding ESTs was clearly enriched in the top 10% of the most abundant ESTs in actively growing *B. sudeticus* cells (i.e., 9.9-fold and 5.7-fold above background; p-value < 0.001), indicating that protein synthesis is highly active in both cytoplasm and chloroplast/ mitochondrial to support growth of *B. sudeticus* cells.

We subsequently examined the relative occurrence density of the eRP- and aRP-encoding ESTs based on ranks by ratio between levels in ND and nitrogen-replete cells. Occurrence density of the eRP-encoding ESTs was found to be enriched in the range containing the middle half and the second half of the eRP-encoding ESTs (i.e., 3.24-fold and 2.29-fold above background; p-value < 0.001), suggesting that change in transcription levels of eRP-encoding ESTs is consistent during response to ND (Figure 3C). On the other hand, occurrence density in all 3 ranges of aRP-encoding ESTs was not enriched. This result supports our hypothesis that co-transcriptional regulation of genes localized in a single genome is relatively easy to achieve compared to those located in different genomes. We noted that transcription of RP genes was not significantly downregulated in *B. sudeticus* cells 3 days after ND (Figure 3D).

### Energy metabolism-associated ESTs appear to be most abundant and highly coherent in actively growing *B. sudeticus* cells

**Figure 4:**
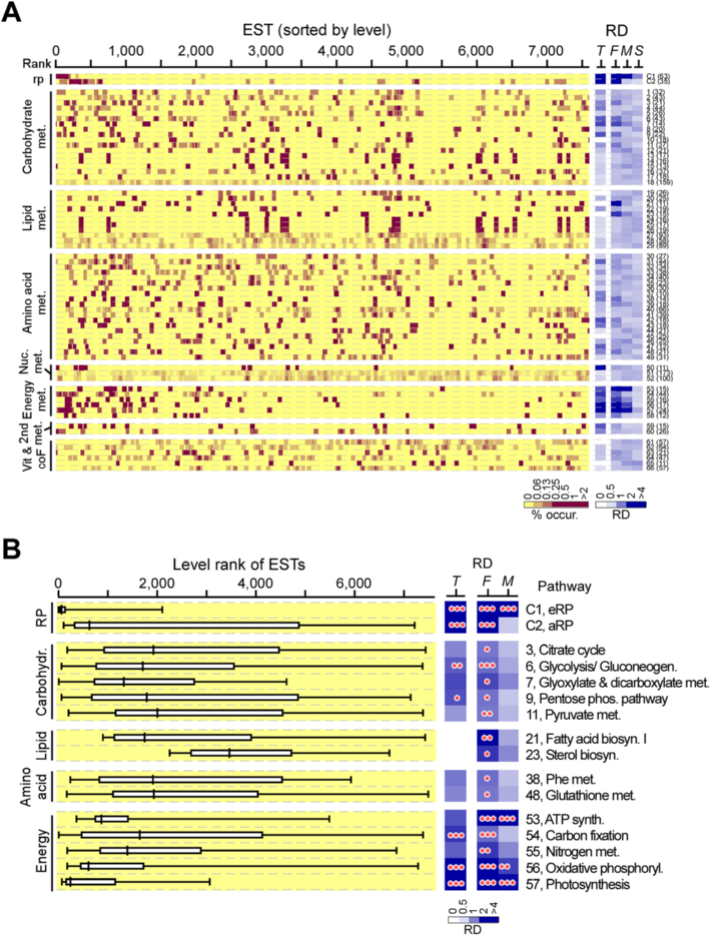
**Coherence of transcription levels in various metabolic pathways in rapidly growing** ***B. sudeticus*** **cells. (A)** Distribution of pathway-associated ESTs based on rank by level. Left panel shows the heat-map of individual EST occurrence based on rank by level. Classes of metabolic pathways are indicated on the far left. Numbers of ESTs associated with the pathway is shown in parentheses. RP stands for ribosomal proteins as control (e.g., C1 or C2). Right panel shows the relative occurrence density in the 3 half-ranges (F for first 50%, M for middle 50%, and S for second 50%) of the pathway-associated ESTs and in the range of top 10% of the most abundant ESTs (i.e., T). Metabolic pathways are numbered on the far right. **(B)** Coherently transcribed metabolic pathways in rapidly growing *B. sudeticus* cells. Distribution of metabolic pathway-associated ESTs displaying the enriched level (i.e., 2-fold above background; p-value < 0.05) in one of the 3 half-ranges (F, M, or S) is shown. Level of statistical significance is indicated by triple asterisk (***), double asterisk (**), and single asterisk (*) for p-value < 0.001, 0.01, and 0.05, respectively.

We examined the consistency in levels of ESTs associated with various metabolic pathways in actively growing *B. sudeticus* cells. To this end, 66 KEGG metabolic pathways were found to associate with at least 10 ESTs based on annotation of the *B. sudeticus* transcriptome (Additional file 5: Table S4). The eRP- or aRP-encoding ESTs were included as control. Relative occurrence density of individual metabolic pathways in ranges containing the first, middle, and second halves of the pathway-associated ESTs based on ranks by level was determined (Figure 4A). Fourteen out of the 66 metabolic pathways exhibited high relative occurrence density in one of the 3 ranges of the pathway-associated ESTs based on ranks by level (i.e., 2-fold above background; p-value < 0.05), 5 of which were found to be enriched in the top 10% of the most abundant ESTs (i.e., 2-fold above background; p-value < 0.05).

Coherent transcription was found in 5 carbohydrate, 2 lipid, 2 amino acid, and 5 energy metabolic pathways (Figure 4B). Of the 14 coherently transcribed pathways, 5 were enriched in the top 10% of the most abundant ESTs: 2 involved in carbohydrate metabolisms (glycolysis/ gluconeogenesis and pentose phosphate pathway) and 3 involved in energy metabolisms (carbon fixation, oxidative phosphorylation, and photosynthesis). Glycolysis is the process of converting glucose into pyruvate and generating small amounts of ATP (energy) and NADH (reducing power) molecules; and the pentose phosphate pathway is a process of glucose turnover that produces NADPH and pentoses (for nucleic acid) [30]. Both were primary metabolism and essential for cell growth. Photosynthesis (i.e., light-dependent photosynthetic reaction) is the process of harvesting light energy and generating ATP and NADH which in turn utilized to reduce carbon dioxide to organic molecules in a process known as carbon fixation (or light-independent photosynthetic reaction). Oxidative phosphorylation is the process of transferring electrons to oxygen and generating ATP in mitochondria. Majority ATP in cells were generated through oxidative phosphorylation [30]. Hence, we concluded that photosynthesis and energy metabolisms are the most active functions required for rapidly growth of *B. sudeticus*.

### Transcription of photosynthetic ESTs is most drastically downregulated upon ND in B. sudeticus

**Figure 5:**
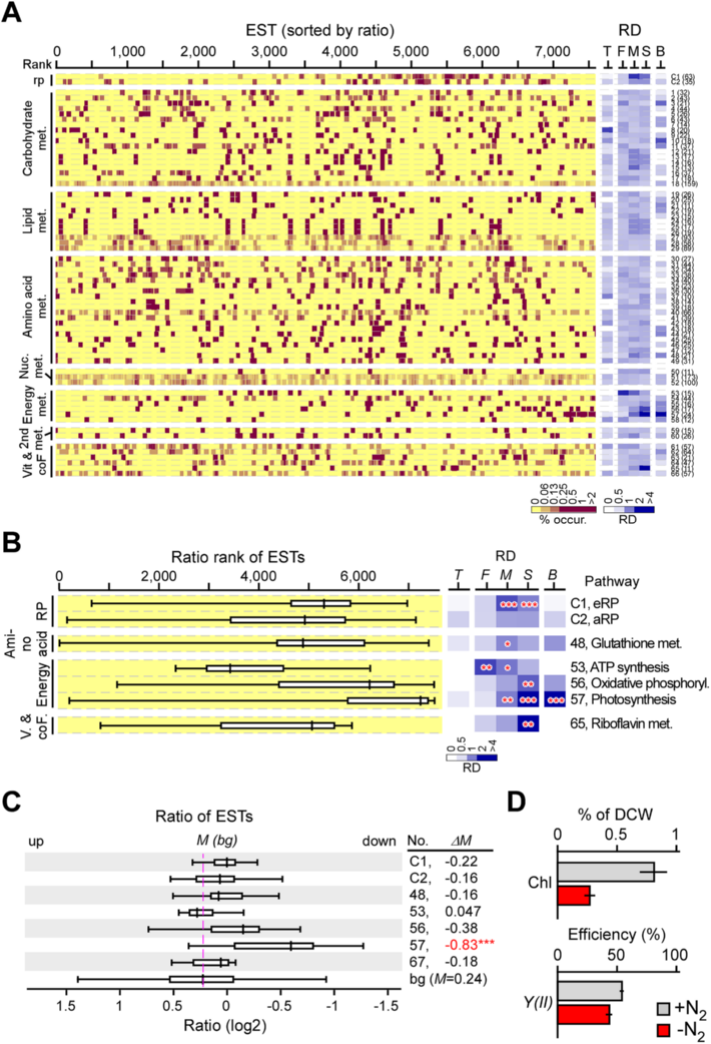
**Coherence of transcriptional alterations in various metabolic pathways in** ***B. sudeticus*** **cells following ND. (A)** Distribution of pathway-associated ESTs based on rank by ratio. The display is identical to Figure 4A. Relative occurrence density in the top10 % and bottom 10% of the most differentially transcribed ESTs is denoted as T and B, respectively. **(B)** Coherently responsive metabolic pathways in *B. sudeticus* cells following ND. The display is identical to Figure 4B. **(C)** Distribution of pathway-associated EST ratios. The display is identical to Figure 3D. Asterisk indicates the level of statistical significance. **(D)** Levels of chlorophyll content (upper panel) and efficiency of quantum yield of photosystem II (bottom panel).

To investigate metabolic pathways that exhibited co-transcriptional alteration in response to ND, the relative occurrence density of the 66 metabolic pathways in the 3 ranges (i.e., the first, middle, and second halves) of the pathway-associated ESTs was determined based on ranks by ratio between levels in ND and nitrogen-replete cells (Figure 5A). Five out of the 66 metabolic pathways such as glutathione metabolism, ATP synthesis, oxidative phosphorylation, photosynthesis, and riboflavin metabolism exhibited coherent response to ND (Figure 5B). It was interesting that 4 of the 5 coherently responsive metabolic pathways displayed a consistency in constitutive transcription (see Figure 4B). On the other hand, of the 5 metabolic pathways exhibiting coherent response, only photosynthesis showed to be downregulated by 43.8% based on the median of photosynthesis-associated ESTs following ND (i.e., p-value < 0.001) (Figure 5C). Median level of the remaining 4 pathways was hardly altered. Photosynthesis was the only pathway whose associated ESTs were enriched in the top 10% of the most downregulated ESTs, indicating that photosynthesis is the most drastically downregulated pathway in *B. sudeticus* following ND.

Consistent with the downregulation of photosynthesis-associated ESTs, chlorophyll contents (i.e., chl a and b) in ND cells were also greatly reduced by 3.1-fold compared to that of nitrogen-replete cells (i.e., 0.26±0.03% versus 0.81±0.11% of CDW, p-value = 0.001) (Figure 5D, upper panel). We subsequently determined the relative quantum yield or efficiency of photosystem II (i.e., Y(II) = (Fm’-F)/Fm’, see Methods), a measurement that is directly related to the quantum efficiency of carbon assimilation or photosynthesis [31]. Interestingly, photosynthesis efficiency was only reduced by 20% upon ND compared to nitrogen-replete conditions (i.e., 0.43±0.01 versus 0.54±0.01) (Figure 5D, bottom panel), not markedly reduced as seen in levels of chlorophyll content. Marginal reduction of quantum yield is consistent with the observation that cell mass increase and carbon assimilation were only slightly affected by ND stress (see Figure 1E). These results implied that photosynthetic components such as light harvesting proteins are in excess under optimal growth conditions. These proteins would be recycled for synthesis of new proteins required for gametogenesis and zygospore formation under ND conditions.

We investigated pathway-associated individual ESTs whose levels were greatly upregulated upon ND. From the 1,185 ESTs that were associated with 68 pathways, we found that 30 involved in 34 pathways were upregulated by 3-fold or greater (Additional file 6: Figure S1). Two lipid metabolism-specific enzymes, monoacylglycerol lipase (EC 3.1.1.23) and 3-oxoacyl-[acyl-carrier-protein] reductase (EC 1.1.1.100) were found. They might play a role in TAG accumulation upon ND.

### Abundant and coherent transcription of energy metabolic genes is conserved in actively growing *B. sudeticus* and *C. reinhardtii* cells

**Figure 6:**
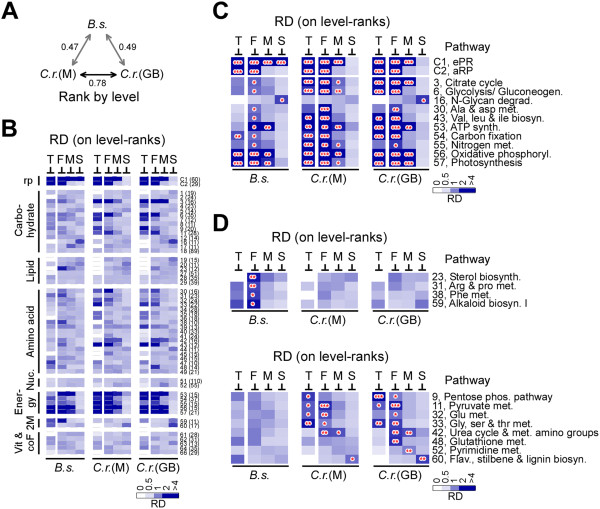
**Coherently transcribed metabolic pathways in rapid growing** ***B. sudeticus*** **or** ***C. reinhardtii*** **cells. (A)** Correlation between EST levels based on rank by level in *B. sudeticus* (i.e., Bs) and *C. reinhardtii* by Miller et al., (i.e., Cr-M) and by Gonzalez-Ballester et al. (i.e., Cr-GB). Correlation coefficient is shown. **(B)** Relative occurrence density of various pathway-associated ESTs based on rank by level in *B. sudeticus* and *C. reinhardtii*. The display is identical to the right panel in Figure 4A. **(C)** Common consistently transcribed pathways in *B. sudeticus* and *C. reinhardtii*. The display is identical to right panel in Figure 4B. Datasets based on *B. sudeticus* (Bs) and *C. reinhardtii* by Miller et al. (Cr-M) and Gonzalez-Ballester et al. (Cr-GB) are indicated. **(D)** Unique consistently transcribed pathways in *B. sudeticus* or *C. reinhardtii*. The display is identical to **(C)**.

We wanted to investigate if the basal transcription of energy metabolic genes was abundant and coherent in the model green microalga *C. reinhardtii*. For this reason, we obtained the transcription profiles of *C. reinhardtii* generated using Illumina technologies in the studies by Miller et al. and Gonzales-Ballester et al. [7, 12]. Based on the best hits in *C. reinhardtii*, there were 4,148 *B. sudeticus* ESTs whose best hits (or homologs) were available in transcriptomic datasets of *C. reinhardtii*[7, 12] (Additional file 7: Table S5). By using the common set of 4,148 ESTs, we found that correlation between basal transcription profiles in *B. sudeticus* and *C. reinhardtii* was moderate (i.e., correlation coefficient = 0.47 ~ 0.49). The correlation between the two independent experiments in *C. reinhardtii* was relatively high (i.e., correlation coefficient = 0.76), indicating a good quality of the two datasets (Figure 6A).

Based on the set of 4,148 ESTs, 53 metabolic pathways had 10 or more associated ESTs (i.e., due to the reduced number of ESTs, 13 pathways were filtered off from the list of 66 pathways based on the set of 7,625 ESTs; see Figures 4 and 5). We found 14 metabolic pathways and ribosomes (i.e., eRP and aRP) showed coherent transcription in rapidly growing log-phase *B. sudeticus* cells (Figure 6B; Additional file 8: Table S6; Additional file 9: Table S7). Though 5 of the 14 pathways were not previously shown when a set of 7,625 ESTs were used (see Figure 4B), the coherent pathways with high level of statistical significance (i.e., p-value < 0.001) remained unchanged.

To ensure a fair comparison, we applied a common set of 4,148 ESTs in *B. sudeticus* and *C. reinhardtii*. The relative occurrence density of the 53 metabolic pathways in 3 ranges (i.e., the first, middle, and second halves of the pathway-associated ESTs) was determined based on rank by level using the common set of 4,148 ESTs in *B. sudeticus* and *C. reinhardtii* (Figure 6B; Additional file 6: Figure S1).

Comparative analysis showed that eRP- and aRP-encoding ESTs were both abundant and coherent in *B. sudeticus* and *C. reinhardtii* (Figure 6C). Furthermore, we found that there were 10 metabolic pathways that were coherent in *B. sudeticus* and *C. reinhardtii*. Of those, 3, 2, and 5 involved in carbohydrate metabolisms, amino acid metabolisms, and energy metabolisms, respectively. We noted that 3 out of the 5 energy metabolic pathways were abundant in *B. sudeticus*. On the other hand, all 5 energy metabolic pathways were abundant (i.e., in one of the two experiments) in *C. reinhardtii*. In addition, 2 carbohydrate and 1 amino acid metabolic pathways were coherent and abundant in *C. reinhardtii*. Coherent pathways tended to be abundant in *C. reinhardtii*, but not in *B. sudeticus*.

We noted that 4 pathways were coherent in *B. sudeticus* but not in *C. reinhardtii* (Figure 6D)*.* On the other hand, 8 were coherent in *C. reinhardtii* but not in *B. sudeticus*, 4 of which were also abundant. Interestingly, all coherent pathways were found to be involved in energy, carbohydrate, and amino acid metabolisms, but not in lipid metabolisms except for sterol biosynthesis in *B. sudeticus*.

### Transcriptional alteration of metabolic pathways but not individual ESTs is correlated in *B. sudeticus* and *C. reinhardtii* following ND

**Figure 7:**
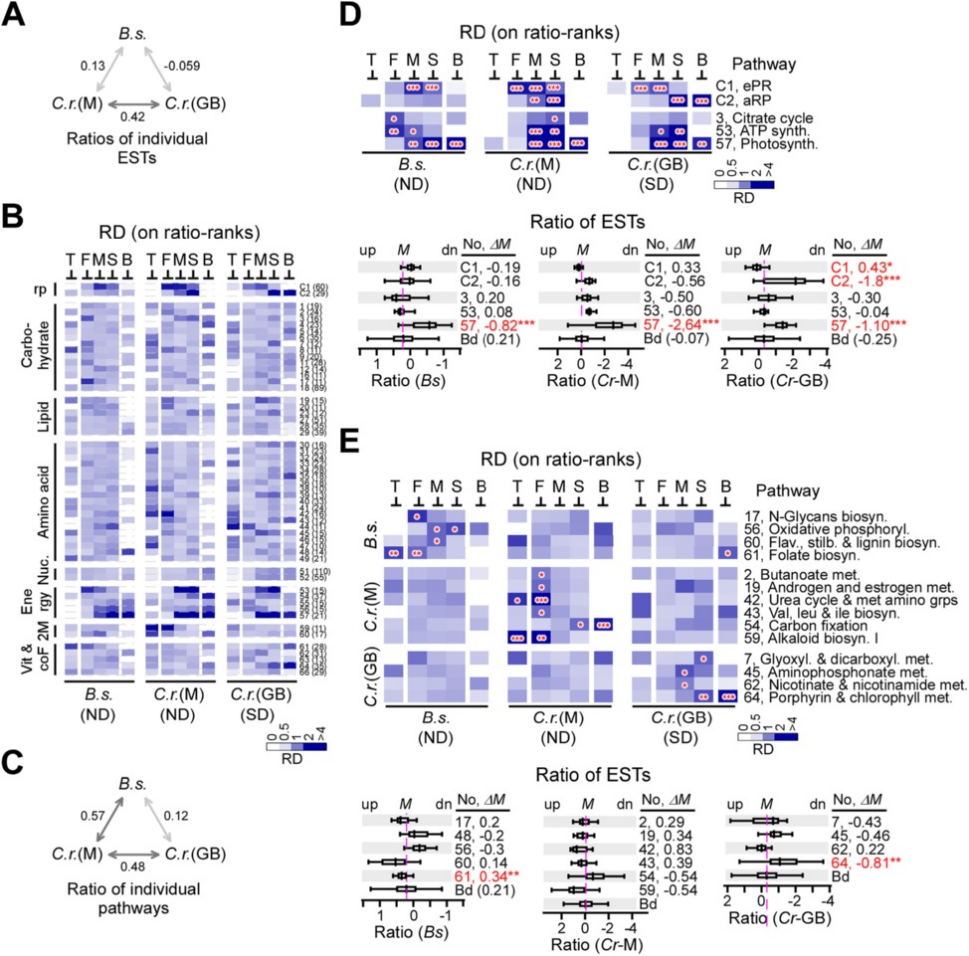
**Coherently responsive metabolic pathways in** ***B. sudeticus*** **or** ***C. reinhardtii*** **following nutrient deprivation. (A)** Correlation between EST ratios in *B. sudeticus* (i.e., Bs) and *C. reinhardtii* (i.e., Cr-M) following ND and *C. reinhardtii* (i.e., Cr-GB) following sulfur deprivation (or SD). Correlation coefficient based on EST ratios is shown. **(B)** Relative occurrence density of various pathway-associated ESTs based on rank by ratio. The display is identical to the right panel in Figure 5A. **(C)** Correlation between median ratios of various metabolic pathway-associated ESTs based on rank by ratio in *B. sudeticus* and *C. reinhardtii* following nutrient deprivation. **(D)** Common consistently responsive pathways in *B. sudeticus* and *C. reinhardtii* following ND. The display of the upper and bottom panels is identical to right panel in Figure 5B and Figure 5C, respectively. **(E)** Unique consistently responsive pathways in *B. sudeticus* and *C. reinhardtii* following nutrient deprivation. The display is identical to **(D)**.

Unlike the absolute level of ESTs, the relative level (or ratio between levels in ND and nitrogen-replete cells) of ESTs in *B. sudeticus* showed no correlation (i.e., correlation coefficient = 0.13) with that of *C. reinhardtii* following ND based on the common set of 4,148 ESTs (Figure 7A). On the other hand, a moderate correlation (i.e., correlation coefficient = 0.42) was observed between EST ratios in *C. reinhardtii* following ND and sulfur deprivation, suggesting that the global transcriptional response to ND was, to some extent, species-specific in green microalgae.

To compare the ND-induced transcription of ESTs associated with various metabolic pathways in *B. sudeticus* and *C. reinhardtii*, we determined the relative occurrence density in the 3 half-ranges of the pathway-associated ESTs based on rank by ratio (Figure 7B). We noted that median ratio of the pathway-associated ESTs in *B. sudeticus* was clearly correlated with that of *C. reinhardtii* following ND but not sulfur deprivation (Figure 7C). This result suggested that transcriptional response of metabolic pathways rather than individual ESTs was conserved in green microalgae *B. sudeticus* and *C. reinhardtii* following ND.

In *B. sudeticus*, only eRP-ESTs but not aRP-ESTs showed coherent response to ND (Figure 7D). On the other hand, both eRP- and aRP-ESTs exhibited coherent response in *C. reinhardtii*. We noted that none of them displayed a significant alteration in transcriptional levels (based on the media) following ND in *B. sudeticus* and *C. reinhardtii*. Interestingly, though, the median level of eRP- or aRP-ESTs was significantly altered in *C. reinhardtii* following sulfur deprivation.

We found that there were 7 metabolic pathways exhibited consistent response (i.e., occurrence density in 1 of 3 half-ranges > 2-fold; p-value < 0.05) in *B. sudeticus* following ND, of which, 3 (i.e., citrate cycle, ATP synthesis, and photosynthesis) were also found to showed coherent response in *C. reinhardtii* in response to ND. While the median level of citrate cycle and ATP biosynthesis was not significantly changed, the median level of photosynthesis was obviously downregulated in *B. sudeticus* and *C. reinhardtii* following ND. In fact, it was also greatly downregulated in *C. reinhardtii* following sulfur deprivation. Consistent with it, aRP- but not eRP-ESTs were also downregulated in *C. reinhardtii* following sulfur deprivation. These results suggested that transcriptional downregulation of photosynthesis is a common response to nutrient deprivation.

Among the coherent pathways specific to species or stresses (Figure 7E), folate biosynthesis was upregulated (p-value < 0.01) in *B. sudeticus* following ND and porphyrin and chlorophyll metabolism was downregulated (p-value < 0.01) in *C. reinhardtii* following sulfur deprivation. Folate and its derivatives are cofactors of one-carbon metabolism that is required for the biosynthesis of nucleotides and amino acids and upregulation of folate biosynthesis was in agreement with biosynthesis of novel proteins for gametogenesis upon ND. Chlorophyll is critical to photosynthesis. Hence, downregulation of porphyrin and chlorophyll metabolism was consistent with the repression of photosynthetic transcription.

## Discussion

Based on the pathway-based approach, we show that the absolute level of ESTs encoding RPs and energy metabolic pathways such as photosynthesis, carbon fixation, and oxidative phosphorylation are highly coherent and abundant in *B. sudeticus* (see Figure 4 and Figure 6) and *C. reinhardtii* (see Figure 6) by using the analysis of EST occurrence density based on rank by level, implying that protein synthesis and energy metabolisms are the most active functions in the rapidly growing *B. sudeticus* and *C. reinhardtii* cells, possibly in all actively growing green microalgal cells. Interestingly, many coherent carbohydrate and amino acid metabolic pathways in *C. reinhardtii* but not in *B. sudeticus* tended to be abundant, probably reflecting the difference between *B. sudeticus* phototrophic growth and *C. reinhardtii* heterotrophic growth. We noted that almost all of the pathways whose coherence in EST steady-state levels occurred in the first 50% (or the upper half) of the pathway-associated ESTs. It implies that there is a tight regulation of the abundantly expressed transcripts in microalgal cells. It would save energy for optimal function of complex metabolic pathways in microalgae.

ND is one of the most widely used methods for enhancement of storage lipids in green microalgae [4–10]. However, ND induces cell cycle arrest and sexual differentiation and spore formation for survival [11]. In this study, we show that photosynthesis is only metabolic pathway whose transcription is most downregulated in *B. sudeticus* following ND and *C. reinhardtii* following ND or sulfur deprivation (see Figure 7). In *C. reinhardtii* following sulfur deprivation, porphyrin and chlorophyll metabolism and aRP-ESTs are also downregulated. In plant, it is known that photosynthetic genes are downregulated in infected or damaged leaf by biotic agents such as arthropods, fungi, bacteria and viral pathogens [32]. Additionally, accumulation of carbohydrate under sink limitation can lead downregulation of photosynthesis in plant [33]. In unicellular green microalgae, it is not known if downregulation of photosynthesis is partly a result of carbohydrate (e.g., starch) accumulation in chloroplast.

In this study, we show that, upon ND, the level of nitrogen in cells is reduced by 2.5-fold. While transcription of photosynthetic genes is drastically reduced, chlorophyll level is also reduced by 3-fold. On the other hand, under ND conditions, cell mass still increases. Carbon content in ND cells is only decreased by 7% compared to that of nitrogen-replete cells. This is consistent with the observation that photosynthesis efficiency is only reduced by 20%. These data indicate that drastic downregulation of photosynthetic transcripts or chlorophylls is primarily for recycling of nitrogen-rich proteins. It implies that photosynthetic proteins such as light-harvesting proteins are in excess in *B. sudeticus*, similar to large-sized antenna-bearing *C. reinhardtii*. It is found that cells with small-sized antenna in C. reinhardtii are more productive compared to that with large-sized antenna [34].

In yeast, genes encoding RPs and protein synthesis are downregulated in response to a number of stress factors including ND [35, 36]. However, ribosome activity is not abolished. It is required for novel protein synthesis for preparation of sexual differentiation and zygospore formation. In mammalian systems, it has been shown that downregulation of protein synthesis is not mediated through dramatic repression of RP gene transcription but through reduced efficiency of translation [37]. It is possible that translation efficiency is decreased upon ND in green microalgae. This would explain the upregulation of eRP-ESTs in *C. reinhardtii* following sulfur deprivation (see Figure 7D). Downregulation of photosynthesis in algae following ND may provide a pool of nitrogen-rich molecules for synthesis of novel proteins required for adaptation and survival [38]. Given that most green microalgae resemble shade leaves having large-sized antenna, recycling of excessive photosynthetic proteins may be not immediately affect the photosynthesis capacity and carbon assimilation upon ND.

Transcriptional response to ND has been thoroughly studied in diatoms [39–41]. In *Phaeodactylum tricornutum*, carbon assimilation is found to continue after ND [39]. In addition, TAG content but not total lipid content is greatly increased following ND [39]. Noticeable reduction of photosynthesis activity (e.g., 20% of total activity) upon ND has also observed in diatom [42]. These results are very similar to what we have observed in *B. sudeticus* (in this study).

ND induces lipid accumulation in *B. sudeticus* (Figures 1C and 1D) and in *C. reinhardtii* [4, 6, 7, 9]. However, no apparent up-regulation of lipid metabolic pathways based on KEGG database [43] is observed following ND in *B. sudeticus* and *C. reinhardtii*. This is consistent with the notion that regulation of storage lipid accumulation is complex in cells following ND: remodeling of membrane lipids for gametogenesis and accumulation of storage lipids through *de novo* biosynthesis and membrane lipid recycling [7]. Alternatively, regulation of TAG biosynthesis may involve post-translational modification [44]. Comparative analysis of global transcriptional response to ND in other microalgal species should provide insight into underlying molecular mechanisms of storage lipid accumulation following ND. Dissecting pathways responsible for lipid accumulation from those regulating sexual differentiation would allow construction of microalgal strains suitable for biomass and lipid production.

## Conclusions

In this study, we utilized the commercial software CLC Genomics Workbench [16] to assemble the transcriptome without reference genome (see Methods). We show a clear correlation between transcription abundances of homologs in *B. sudeticus* and *C. reinhardtii*, suggesting a decent assembly of *B. sudeticus* transcriptome without reference genome using commercial software (see Figures 6 and 7). It provides a way for biologists to perform transcriptome studies in nonmodel organisms (i.e., no reference genome sequences) without much bioinformatics skills. By using the pathway-based approach, without performing time-course experiments, we are able to identify a number of probable gene regulatory networks (i.e., genes with coherent transcription in a pathway) that are involved in regulation of various metabolic pathways in rapidly growing or ND-induced *B. sudeticus* cells. Our result is useful for construction of motif-specific transcription regulatory networks when genome sequence is available. For transcriptome analysis involving a large numbers of samples such as in a time-course experiment with multiple repeat, it is economical to use DNA microarray-based technologies. Hence, *de novo* assembled transcriptome is also useful in designing DNA microarray.

## Methods

### Algal strains and culture manipulation

The *Botryosphaerella* (or *Botryococcus*) *sudeticus* (UTEX2629) strain was obtained from the Culture Collection of Autotrophic Organisms (http://ccala.butbn.cas.cz/) with a strain catalog number of CCALA780 and the *C. reinhardtii* (UTEX89 or CC1009) strain was obtained from Chlamy Center (http://www.chlamy.org). CC1009 is a cell wall proficient and *NIT1*^*+*^ and *NIT2*^*+*^ strain. The cells were cultivated in 1 L of HS medium [25] or Bold’s modified Bristol (BB) [26] medium in a 2.5 L low-form flask with shaking at 100 rpm at 25°C under continuous illumination of ~250 mol photon m^-2^ sec^-1^. Culture was supplied with 2% CO_2_ through bubbling. BB medium without sodium nitrate (or BB-N) was used as nitrogen-depleted medium.

Cell density of cultures was determined using cell dry weight (CDW) in g/L. Approx. 50 ml cell culture were harvested by filtration using the glass fiber filter GF/A (Whatman/GE Healthcare, Kent, UK) and dried in oven at 80°C overnight. CDW was measured in triplicate using the AG204 balance (Mettler-Toledo Inc., Columbus, OH).

The ratio of flagellates in cultures was determined by counting a total of 500 individual cells (e.g., flagellate or non-flagellate) in at least five independent fields in triplicate using Zeiss Axiovert 200 M microscope (Carl Zeiss AG, Oberkochen, Germany).

To assess flocculation, shaken flasks (Corning Incorporated, Corning, NY, USA) were brought to standstill for 30 min to score for flocculation or sedimentation of cells at the bottom of flasks.

Log-phase culture in BB+N medium at the CDW of ~1.2 g/L is diluted by 8-fold in fresh BB+N and BB-N medium and cultivated for 3 days as nitrogen-replete and nitrogen-depleted cultures, respectively.

### Elemental analyses

To determine nitrogen and carbon content of cells, approx. 3mg dry cell powder were subjected to elemental analysis using the the Vario Micro Cube (Elementar Analysensysteme GmbH, Hanau, Germany) in the Elemental Analysis Laboratory, Department of Chemistry, National University of Singapore. To determine nitrate in medium, 2 ml culture supernatant was subjected to OD measurement using the Spectroquant Test Kit for Nitrate (Merck KGaA, Darmstadt, Germany) according to the manufacturer’s instruction.

### Analyses of chlorophyll fluorescence and PAM-fluorescence of photosynthesis

To determine chlorophyll content, cells from 2 ml culture were harvested by centrifugation. The resulting cells were resuspended in 96% ethanol and broken in the glass-bead beater FastPrep homogenizer (MP Biomedicals, Solon, OH, USA). After incubation with ethanol for 2 h at 4°C, cell debris was cleared by centrifugation. The resulting supernatant was subjected to OD measurement at the wavelength of 645 nm and 663 nm, with the 96% ethanol as blank. Total chlorophyll content (i.e., chl a and b) was estimated using a formula C (mg/L) = 20.2OD_645_+8.05D_663_[45] and was converted to % of CDW.

To determine photosynthesis efficiency, cells were subjected to PAM-fluorescence analysis using the fluorometer Imaging-PAM (Heinz Walz GmbH, Effeltrich, Germany) according to the manufacturer’s instruction. Briefly, the Fm’ and F were determined at the arctic light of 250 µmol photon m^-2^ sec^-1^ to mimic the growth conditions. The quantum yield was based on the formula Y(II) = (Fm’-F)//Fm’ [46].

### Fluorescence microscopic analyses and fluorescence-activated cell sorting (FACS) analysis

To examine the lipid content in *B. sudeticus* cells, 4 μl of 0.25 mg/ml Nile red in acetone was directly added to 1 ml of fresh culture. After incubation at RT in dark for 15 min, fluorescence signals in *B. sudeticus* cells were examined using the Zeiss Axiovert 200 M (Carl Zeiss AG, Oberkochen, Germany) with a Zeiss EC Plane-Neofluar 40x/0.75 objective lens. The images were captured by a CoolSNAP HQ monochrome digital camera (Roper Scientific, Ottobrunn, Germany) and processed using MetaMorph software (Molecular Devices, Sunnyvale, CA).

*B. sudeticus* cells were fixed with 10% formaldehyde (w/v) at RT for 15 min. The cells were washed twice with 1×PBS and stained by Nile red at the final concentration of 1 μg/ml for 15 min in dark. In microscopic analysis, Nile red stained cells were examined using the Zeiss Axiovert 200 M (Carl Zeiss AG, Oberkochen, Germany) with A Zeiss filter *cube no. 15* (*EX BP 546/12*, BS FT 580, EM LP 590). In FACS analysis, Nile red stained cells were subjected to the analysis of BD FACS Calibur flow cytometer (Becton Dickinson Biosciences, San Jose, CA).

### TLC and GC-MS analysis

Total lipid was exacted with methanol/ chloroform (2:1 by volume) solution and was quantified via gravimetric analysis after evaporation of solvents. Then it was dissolved in chloroform at a concentration of 0.1 mg/µl. Equal amount of lipid was loaded on silica TLC plate (60F254, Merck Corporate, Whitehouse Station, NJ, USA) and developed in hexane/ diethyl ether/ acetic acid (35:15:0.1 by volume). TAG (tri-oleic acid (C18:1, [cis-9]) glyceride) and FFA (oleic acid (C18:1, [cis-9])) (Sigma-Aldrich, St. Louis, MO) was used as standard. Lipid profile on TLC plate was developed using iodine vapor. TAG and FAA contents were estimated through densitometric analysis of TLC profile. Hydrocarbons were exacted in equal amounts of total lipids with hexane. Hydrocarbons were analyzed using Shimadzu GC/MS-QP2010 Plus system (Shimadzu, Kyoto, Japan) equipped with a HP-5ms Ultra Inert column (30 m × 0.25 mm × 0.25 μm, Agilent Technologies, Santa Clara, CA).

### Construction of cDNA libraries for next-generation sequencing analysis

Total RNA was extracted from *B. sudeticus* cells using TRIzol® Plus RNA Purification System (Invitrogen-Life Technologies Co., Carlsbad, CA, USA) according to manufacturer’s protocol. Appr. 4 µg of the resulting total RNA was used for synthesis of cDNA using the TruSeq RNA Sample Prep Kit (Invitrogen-Life Technologies Co.) according to manufacturer’s instruction including synthesis of first and second strands cDNA, end repair, 3’-end adenylation, adapter ligation, fragment enrichment (e.g., ~260 bps in length), and library validation, quantification, and quality assessment with a bioanalyzer (Agilent Technologies; Santa Clara, CA, USA). The libraries are sequenced using the Illumina HiSeq 2000 Sequencer (BGI, Shenzhen, China).

### *De novo* assembly of Illumina short sequence reads

Over 2 Giga-base clean paired-end reads (90 bps in length/ read) from each library were generated using HiSeq2000 technology. A total of 56 million reads from both growth conditions were pooled and subjected to de novo assembly using the CLC Genomics Workbench software (CLC Bio, Swansea, UK). As a result, 56 thousand nonredundant contigs/ scaffolds/ ESTs ranging from 200 to 15,500 bps in length were obtained. More than 90% of total reads from each growth condition were mapped back to the 56 thousand ESTs, ~99% of which were found in both conditions. Read hits per EST were normalized to RPKM (Reads Per Kilobase per Million mapped reads) for estimation of ESTs transcription levels.

### Annotation of the *B. sudeticus* transcriptome

All nonredundant ESTs were subjected to sequence homology comparison using the Basic Local Aliment Search Tool BLASTX suit against the “best” proteins in the model green microalgae *C. reinhardtii* and *C. variabilis* (genome.jgi-psf.org/Chlre4 and genome.jgi-psf.org/ChlNC64A_1). A total of 6,559 ESTs showed to have a best hit (i.e., expectation value < 1E-06) in the *C. reinhardtii* “best” proteins. Among the remaining ESTs, 1,066 showed to have a best hit (i.e., expectation value < 1E-06) in the *C. variabilis* “best” proteins. To this end, 7,625 ESTs (13.6%) in *B. sudeticus* were annotated based on the best hits in *C. reinhardtii* and *C. variabilis* (Additional file 2 Table S1). Of 7,625 ESTs, 4,860 were found to be associated with at least one of the Gene-ontology (GO) terms and 1,129 were associated with at least one of the Kyoto Encyclopedia of Genes and Genomes (KEGG) functions.

### Analysis of relative occurrence density of the pathway-associated ESTs

Based on EST annotations, 66 (or 53) metabolic pathways were found to have 10 or more pathway-associated ESTs by using the set of 7,625 (or 4,148) ESTs (Additional file 5: Table S4). Average occurrence density (or background) of the pathway-associated ESTs is calculated as the ratio between numbers of pathway-associated ESTs and all ESTs tested. Occurrence density in the range containing first 50%, middle 50%, or second 50% of the pathway-associated ESTs is calculated as the ratio between 50% of the pathway-associated ESTs and all ESTs within the respective range. Relative occurrence density in a particular range is the ratio between the occurrence density in that range and the background.

Coherence of the constitutive transcription levels (or ND-induced transcription ratios) of various metabolic pathway-associated ESTs is defined as those whose relative occurrence density in one of the 3 half-ranges based on rank by level (or ratio) is 2-fold above background and p-value < 0.05. We assessed whether the coherent pathways were the most abundant (or most differently transcribed) ones in *B. sudeticus* by using the relative occurrence density in the top 10% of the most abundant (or most differentially transcribed) ESTs based on rank by level (or ratio) (i.e., >2-fold above background; p-value < 0.05).

The distribution of pathway-associated ESTs based on ranks (i.e., level or ratio) in heat-map display is binned by an average sliding window of 11 consecutive ranks. The entire distribution of the ESTs in each pathway is normalized to 1 or 100%.

### Comparative analysis between *B. sudeticus* and *C. reinhardtii*

Of 7,625 annotated ESTs in *B. sudeticus*, 6,559 have a best hit in the *C. reinhardtii* “best” proteins. Based on the transcriptome analysis by Miller et al. [7] and Gonzalez-Ballester et al. [12], 4,148 out of 6,559 ESTs were available for comparisons. Therefore, coherence of transcriptions of 53 metabolic pathways was determined based on the set of 4,148 ESTs (Additional file 8: Table S6).

### Statistical analysis

Binomial test was used to determine the statistical significance of enrichment for a number of pathway associated ESTs (i.e., observed) that are present within a range of ranked ESTs (i.e., trials). Average occurrence (or background level) of a pathway associated ESTs is the ratio between numbers of the pathway associated ESTs and all ESTs tested.

T-test was used to determine the statistical significance of level changes of pathway associated ESTs compared to all ESTs tested (or background). P-values are subjected to Bonferroni correction according to numbers of individual pathways tested against background.

The raw HiSEQ2000 pair-end sequencing data used in this study are available at the NCBI’s Sequence Read Achieve (http://www.ncbi.nlm.nih.gov/sra) with the SRA Study accession number, SRA026047.

## Acknowledgements

The authors would like to thank P. Datta, J. Jeyakani, and N. Clark for assistances and advices on *de novo* transcriptome assembly. The authors are grateful to K. Nelson of JCVI and the anonymous reviewers for their comments that have greatly improved the manuscript. This work is partly supported by the 7^th^ JCO/A-STAR grant 11/03/FG/07/06 to JL and YC and the 8^th^ JCO/A-STAR 1131CFG008 to JL. JZ, an undergraduate of Sichuan University, Chengdu, China, is an attachment student in GIS; LF and DS are supported by the post-doctoral fellowships under the JCO Grants 11/03/FG/07/06 and 1131CFG008, respectively.

## Electronic supplementary material

Additional file 1: Table S8: Characterizations of cell cultures supplemented with or without nitrogen. (XLSX 13 KB)

Additional file 2: Table S1: List of 7,625 annotated ESTs in B. sudeticus. (XLS 2 MB)

Additional file 3: Table S2: List of EST-associated GO terms. (XLS 414 KB)

Additional file 4: Table S3: List of EST-associated KEGG metabolic pathways. (XLS 44 KB)

Additional file 5: Table S4: List of 66 metabolic pathways and occurrence densities based on 7,625 ESTs. (XLS 38 KB)

Additional file 6: Figure S1: Upregulated (>3-fold) pathway-associated ESTs. (PDF 96 KB)

Additional file 7: Table S5: List of 4,148 ESTs in B. sudeticus and C. reinhardtii. (XLS 1 MB)

Additional file 8: Table S6: List of 53 metabolic pathways and occurrence densities based on 4,148 ESTs. (XLS 54 KB)

Additional file 9: Table S7: Occurrence of genes based on ranks in various pathways. (XLSX 2 MB)
