# Supplementary material for: De novo transcriptome profiling uncovers a drastic downregulation of photosynthesis upon nitrogen deprivation in the nonmodel green alga Botryosphaerella sudeticus
Source: BMC Genomics. 2013 Oct 19;14(1):715. doi: 10.1186/1471-2164-14-715 (PMC4050207; doi:10.1186/1471-2164-14-715)
Supplement: Supplementary file 6 — Additional file 6: Figure S1: Upregulated (>3-fold) pathway-associated ESTs. (PDF 96 KB) [file 12864_2012_6099_MOESM6_ESM.pdf]

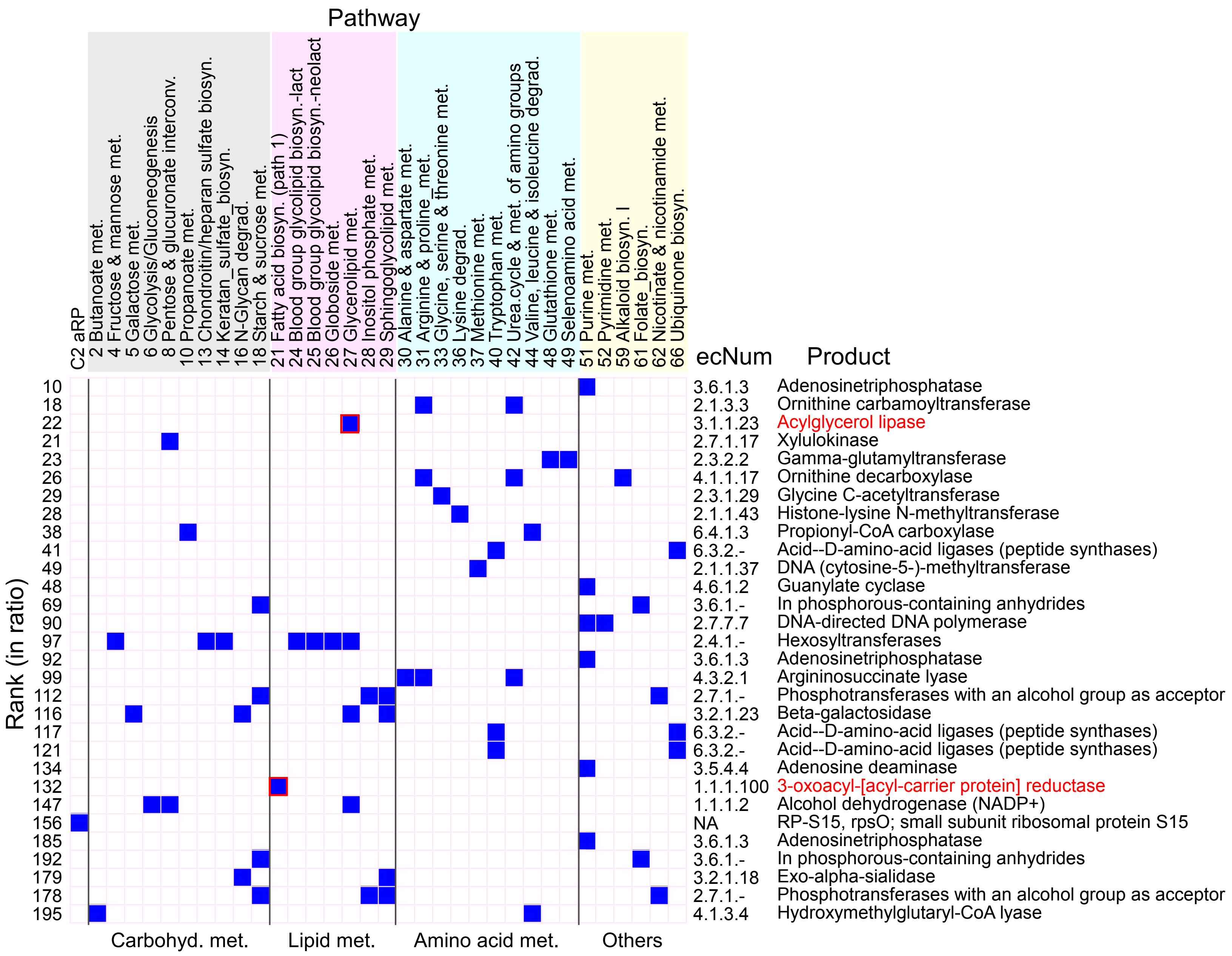

Figure S1. Upregulated (>3-fold) pathway-involving ESTs. Row indicates EST whose rank by ratio is shown on the left and enzymatic activity is shown on the right. Column indicates metabolic pathways. Red boxed ESTs are unique to lipid metabolism.
